# *EPS8L2* is a new causal gene for childhood onset autosomal recessive progressive hearing loss

**DOI:** 10.1186/s13023-015-0316-8

**Published:** 2015-08-19

**Authors:** Malika Dahmani, Fatima Ammar-Khodja, Crystel Bonnet, Gaelle M. Lefèvre, Jean-Pierre Hardelin, Hassina Ibrahim, Zahia Mallek, Christine Petit

**Affiliations:** Equipe de Génétique, Laboratoire de Biologie Cellulaire et Moléculaire, Faculté des Sciences Biologiques, Université des Sciences et de la Technologie Houari Boumédiène (USTHB), El Alia, Bab-Ezzouar, Algiers, Algeria; Syndrome de Usher et autres Atteintes Rétino-Cochléaires, Institut de la vision, 75012 Paris, France; UMRS 1120, Institut National de la Santé et de la Recherche Médicale (INSERM), Paris, France; Sorbonne Universités, UPMC Université Paris 06, Complexité du Vivant, Paris, 75252 Cedex 05 France; Unité de Génétique et Physiologie de l’Audition, Institut Pasteur, 75015 Paris, France; Service d’otorhinolaryngologie, Centre Hospitalier Universitaire Mustapha Pacha, Algiers, Algeria; Service d’otorhinolaryngologie, Centre Hospitalier Universitaire Bab El Oued, Algiers, Algeria; Collège de France, 75005 Paris, France

**Keywords:** Epidermal growth factor receptor pathway Substrate 8 L2 (EPS8L2), Progressive deafness, Whole-exome sequencing, Stereocilia bundle

## Abstract

**Background:**

More than 70 % of the cases of congenital deafness are of genetic origin, of which approximately 80 % are non-syndromic and show autosomal recessive transmission (DFNB forms). To date, 60 DFNB genes have been identified, most of which cause congenital, severe to profound deafness, whereas a few cause delayed progressive deafness in childhood. We report the study of two Algerian siblings born to consanguineous parents, and affected by progressive hearing loss.

**Method:**

After exclusion of *GJB2* (the gene most frequently involved in non-syndromic deafness in Mediterranean countries), we performed whole-exome sequencing in one sibling.

**Results:**

A frame-shift variant (c.1014delC; p.Ser339Alafs*15) was identified in *EPS8L2*, encoding Epidermal growth factor receptor Pathway Substrate 8 L2, a protein of hair cells’ stereocilia previously implicated in progressive deafness in the mouse. This variant predicts a truncated, inactive protein, or no protein at all owing to nonsense-mediated mRNA decay. It was detected at the homozygous state in the two clinically affected siblings, and at the heterozygous state in the unaffected parents and one unaffected sibling, whereas it was never found in a control population of 150 Algerians with normal hearing or in the Exome Variant Server database.

**Conclusion:**

Whole-exome sequencing allowed us to identify a new gene responsible for childhood progressive hearing loss transmitted on the autosomal recessive mode.

## Background

Deafness is the most common sensory deficit in humans, with an incidence of 1 in 700 live births. It is estimated that about two thirds of prelingual severe to profound isolated (non-syndromic) deafness cases have a genetic cause in developed countries [1], and autosomal recessive inheritance (DFNB) accounts for 80 % of the genetic cases [2]. Cases with autosomal recessive non-syndromic hearing loss (ARNSHL) are more prevalent in populations where consanguineous marriages are common, including Maghrebi populations. To date, 60 DFNB genes have been identified (http://hereditaryhearingloss.org/). Recessive deafness is usually prelingual, severe or profound, and fully penetrant, but some DFNB genes cause progressive hearing loss with delayed onset in childhood [3, 4].

In Algeria, mutations in *GJB2*, encoding connexin 26 (Gap Junction Protein Beta 2), account for no less than 35 % of the cases [5–7]. Mutations in other DFNB genes have also been identified, which illustrates the ARNSHL genetic heterogeneity in this population [8]. However, the genetic bases of progressive deafness beginning in childhood have not been characterized yet. Here, we report the identification, by whole-exome sequencing (WES), of a new causal gene for recessively inherited progressive deafness in an Algerian family.

## Patients and methods

### Patients

The two affected siblings were recruited in deafness schools of Blida and Baraki in Algiers (Algeria), and clinically examined in the otorhinolaryngology department at Bab El Oued hospital in Algiers. Medical history and physical examination of the patients did not reveal any non-genetic cause for the deafness and confirmed its non-syndromic nature. Hearing thresholds were determined by pure tone audiometry between 125 Hz and 8000 Hz, using air-conduction and bone conduction of sound.

### Methods

The study was approved by the local Ethics Committee and the Committee for the Protection of Individuals in Biochemical Research as required by French legislation. A written consent for genetic testing was obtained from every family member. DNA was extracted from peripheral blood lymphocytes using the Promega Wizard Genomic DNA Purification Kit (Promega, Madison, MI, USA, Cat. # A1120). Whole-exome sequencing and bioinformatic analysis were carried out as previously described [9]. To validate the mutation in *EPS8L2*, sequencing of exon 12 was performed by the Sanger technique with the following primers: EPS8L2-12 F 5′-GTCTGTGCTGAGGGGAGG-3′ and EPS8L2-12R 5′-CTCTCCAGAACTGGCCCAC-3′ (http://bioinfo.ut.ee/primer3-0.4.0/primer3/).

## Results and discussion

**Fig. 1:**
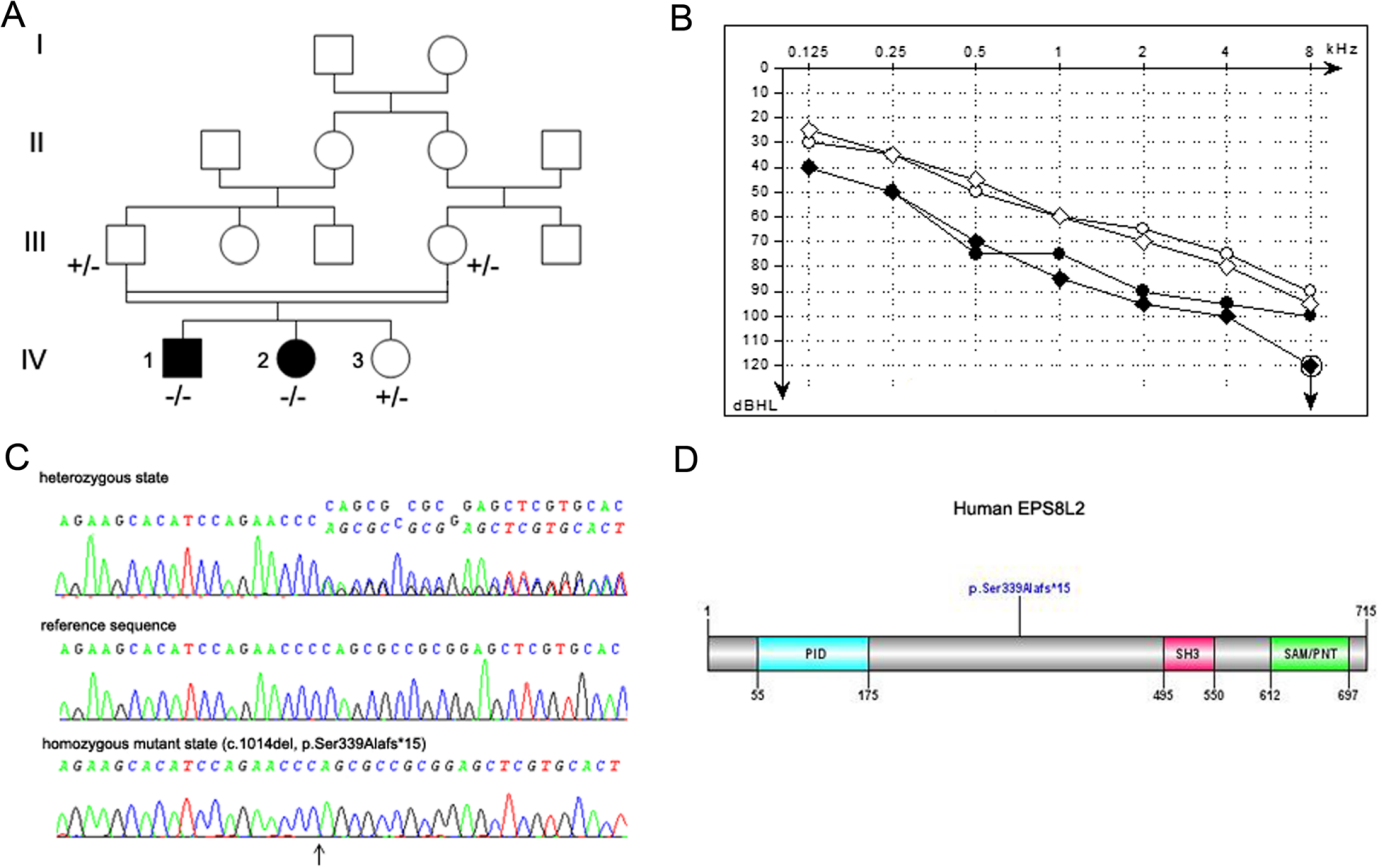
Clinical and molecular data of the patients harboring a biallelic homozygous frameshift mutation in *EPS8L2*. **a** Pedigree showing the segregation of the mutation in the family. The + and – signs denote the wild type and mutant alleles, respectively. The two clinically affected siblings are indicated by black symbols. The double horizontal bar joining the parents in generation III indicates consanguinity. **b** Air-conduction audiometric curves for patient IV.2 at the ages of 6 (open symbols) and 10 (black symbols) years of age. For each pure tone frequency tested (from 0.25 to 8 kHz), the hearing thresholds (in dB HL) for the right and left ears are indicated with circles and diamonds, respectively. **c** DNA sequencing chromatograms showing the mutation (arrow). **d** Schematic representation of the human EPS8L2 protein. The protein (715 amino acids) contains a phosphotyrosine interaction domain (PID), an SRC Homology 3 (SH3) domain, and an effector region (46 % of amino acid sequence identity with F-actin binding domain of EPS8) including a sterile alpha motif/pointed domain (SAM/PNT)

We studied an Algerian family with two siblings (IV.1 and IV.2) presenting with ARNSHI (Fig. 1a). The two affected children were born to normal hearing second-cousin parents, and reportedly had normal hearing for the first 3 years. The first signs of hearing impairment appeared around 4 years of age. By 6 years of age, they presented a bilateral elevation of the hearing threshold, starting at 30 dB at low frequencies (125 Hz) and increasing steadily up to 90 dB at high frequencies (8000 Hz) (Fig. 1b). At the age of 10, the hearing threshold increased further at all frequencies, ranging from 40 dB at 125 Hz, up to 100 dB at 8000 Hz (Fig. 1b and data not shown). Of note, audiograms showing parallel slopes at 6 and 10 years of age are rather unusual.

First, we explored the possibility of mutations in *GJB2* by Sanger sequencing of the non-coding exon and the single coding exon of this gene for each patient, and screened the patients for the common deletion reported in the promoter region [10]. Since no mutations were found, we then proceeded to sequence the whole exome of patient IV.2, and looked for homozygous, compound heterozygous, or heterozygous variants with a predicted pathogenicity. None of the genes known to cause autosomal recessive or autosomal dominant forms of deafness showed any such variants in coding and non-coding exons or splice sites. The *STRC* and *OTOA* genes, which were poorly covered by WES, were further analyzed by single nucleotide polymorphism (SNP) array and q-PCR. No evidence for genomic rearrangement was found. Variants with a prevalence superior to 0.01 % in the DBSNP132, 1000 genomes, HapMap, and Exome Variant Server databases were excluded. Given the parents' consanguinity, the causal mutation was searched preferentially among homozygous nonsense, frame-shift, splice site, and missense variants. From the originally-identified 75,753 SNPs and 5918 insertions/deletions, only 5 SNPs and 1 deletion (Table 1) appeared as good candidates. The four missense variants and the synonymous/splice exon variant were deemed non pathogenic by the prediction algorithms SIFT, PolyPhen2, Mutation Taster and Nnsplice. In contrast, the homozygous 1-bp deletion c.1014delC (p.Ser339Alafs*15), located in exon 12 of the *EPS8L2* gene, was predicted to cause a frame shift leading to premature termination of translation*.* The causal role of this variant was further supported by prior work showing that inactivation of this gene in the mouse caused progressive deafness [11]. After Sanger sequencing of *EPS8L2* exon 12 of the two affected children, the unaffected sister, and the parents, the segregation analysis confirmed that the mutation was homozygous only in the deaf children, whereas the parents and the unaffected sibling were heterozygous for this variant (Fig. 1a, c). Lastly, the variant was never detected in a control population of 150 Algerians with normal hearing or in the Exome Variant Server database. We concluded that this frame-shift mutation, predicted to result either in a truncated inactive protein, or in no protein at all due to nonsense-mediated mRNA decay, is responsible for the progressive hearing loss of the two Algerian patients. *EPS8L2* can thus be added to the list of DFNB genes already reported to cause progressive ARNSHL in humans: *SLC26A4* [12, 13], *MYO3A* [14], *LOXHD1* [15], *PJVK* [16–20], *SERPINB6* [21], *TPRN* [22, 23], *TMPRSS3* [24, 25], *GIPC3* [26], *SYNE4* [27], *GRXCR2* [28], *CLIC5* [29], *TMC1* [30], *GRXCR1* [31], and *LRTOMT* [32]. Of note, about 25 % of the DFNB genes identified so far are responsible for progressive forms of deafness, and several of them (*SLC26A4*, *LOXHD1*, *PJVK*, *TMPRSS3*, *GIPC3*, *TMC1*, *GRXCR1, LRTOMT*) can cause either non-progressive or progressive forms of deafness.

**Table 1:** Analysis of Indel and SNP files showing the variants found in the homozygous state in patient IV.2

Gene name	Refseq	Type of variant	Mutation	Exon #
*ATAD5*	NM_024857.3	missense	c.643G > A; p.Asp215Asn	2
*CDRT1*	NM_006382.3	synonymous/splice	c.1848A > G; p.Lys616Lys	11
*EPS8L2*	NM_022772.3	frame-shift	c.1014delC; p.Ser339Alafs*15	12
*NLRP6*	NM_138329.1	missense	c.2138C > T; p.Ala713Val	5
*OR51V1*	NM_001004760.2	missense	c.631C > G; p.Leu211Val	1
*TOLLIP*	NM_019009.3	missense	c.481G > A; p.Asp161Asn	4

*EPS8L2* has 24 exons and codes for a 715 amino acid protein (Fig. 1d), which is a member of the actin-binding protein EPS8 (epidermal growth factor receptor pathway substrate 8) family. This family includes four members, EPS8 and the three EPS8-like proteins (EPS8L1, EPS8L2, and EPS8L3), with partially overlapping functions [33]. In the mouse ear, EPS8L2 was detected at the stereocilia tips of both cochlear and vestibular hair cells. *Eps8L2* knockout mice undergo progressive hearing loss, as the result of the progressive disorganization of the hair bundles of both inner and outer hair cells. Scanning electron microscopy analysis of the hair bundles in the cochlea of these mice showed that stereocilia of the tall row are shorter and fewer than those of wild-type mice, whereas both the middle and small stereocilia rows seem to be preserved and unaffected [11]. By contrast, mutations in the EPS8 gene cause profound congenital deafness in mice and humans [34, 35]. In the mouse cochlea, *Eps8* is essential for the initial elongation of stereocilia [34, 36], whereas *Eps8L2* is required for their maintenance in mature hair cells [11].

## Conclusion

Deafness is a highly heterogeneous disorder, and many causative genes still remain to be identified. Here, despite several limitations (e.g. only a single family possibly analysed, uneven exon coverage at certain loci by WES), we were able to identify a new pathogenic variant responsible for ARNSHL in two Algerian siblings, by combining the powers of WES and genetics. This report is the first to incriminate *EPS8L2*, a gene formerly known to cause deafness in rodents, as a causal gene for progressive hearing loss in humans.
